# Tiling Assembly: a new tool for reference annotation-independent transcript assembly and novel gene identification by RNA-sequencing

**DOI:** 10.1093/dnares/dsv015

**Published:** 2015-09-03

**Authors:** Kenneth A. Watanabe, Arielle Homayouni, Tara Tufano, Jennifer Lopez, Patricia Ringler, Paul Rushton, Qingxi J. Shen

**Affiliations:** 1 School of Life Sciences, University of Nevada Las Vegas, 4505 South Maryland Parkway, Las Vegas, NV 89154, USA; 2 Texas A&M AgriLife Research, 17360 Coit Road, Dallas, TX 75252, USA

**Keywords:** RNA-sequencing, transcript assembly, *Oryza sativa*, cufflinks, genome annotation, novel genes

## Abstract

Annotation of the rice (*Oryza sativa*) genome has evolved significantly since release of its draft sequence, but it is far from complete. Several published transcript assembly programmes were tested on RNA-sequencing (RNA-seq) data to determine their effectiveness in identifying novel genes to improve the rice genome annotation. Cufflinks, a popular assembly software, did not identify all transcripts suggested by the RNA-seq data. Other assembly software was CPU intensive, lacked documentation, or lacked software updates. To overcome these shortcomings, a heuristic *ab initio* transcript assembly algorithm, Tiling Assembly, was developed to identify genes based on short read and junction alignment. Tiling Assembly was compared with Cufflinks to evaluate its gene-finding capabilities. Additionally, a pipeline was developed to eliminate false-positive gene identification due to noise or repetitive regions in the genome. By combining Tiling Assembly and Cufflinks, 767 unannotated genes were identified in the rice genome, demonstrating that combining both programmes proved highly efficient for novel gene identification. We also demonstrated that Tiling Assembly can accurately determine transcription start sites by comparing the Tiling Assembly genes with their corresponding full-length cDNA. We applied our pipeline to additional organisms and identified numerous unannotated genes, demonstrating that Tiling Assembly is an organism-independent tool for genome annotation.

## 1. Introduction

RNA-sequencing (RNA-seq) technology enables whole-transcriptome profiling via the collection and mapping of short cDNA fragments (reads) to a reference genome.^1,2^ Regions of the genome where many reads align indicate such regions are highly expressed.^3^ Regions where no known gene has been annotated and a large number of reads align are indicative of an undiscovered gene.^4^

Reconstruction of transcripts can be obtained through a variety of computational strategies, each of which has its own benefits and drawbacks. These assembly algorithms fall into two general classes, *ab initio* assembly and *de novo* assembly. *Ab initio*, mapping-first approaches rely on the availability of a reference genome to which the short reads can be aligned.^5^ The major drawback of this method stems from the dependency of accurate transcript identification on the presence of a high-quality reference genome.^6^ The main benefit of *ab initio* assembly is the maximum sensitivity exhibited for gene identification,^5,7^ though higher sensitivity tends to result in a lower accuracy due to a higher number of false-positive genes being reported.^7^ On the other hand, *de novo* transcript assembly, or assembly-first approaches, is independent of a reference genome and directly determines the transcripts of a genome through the short reads.^5^ Since this avenue is dependent solely on short-read data, genes with low read coverage due to low expression levels can result in inaccurate determination of full-length transcripts.^8^ The main benefit provided by *de novo* assembly methods is the lack of reliance on a reference genome, which makes it a vital tool for gene identification in organisms that lack a reference genome. Thus, both genome biology as well as the availability of an accurate and complete genome play major factors in the decision to use *ab initio* assembly versus *de novo* assembly.^7^

Recently, we published a clustering algorithm to identify novel protein- and microRNA-coding genes by searching only the unannotated regions of the rice genome.^9^ However, this method relied on the genome being partially annotated. Analysis of the RNA-seq data with other transcript assembly software revealed that they failed to identify genes, were CPU intensive, or lacked documentation or recent software updates. Here, we report an improved algorithm for *ab initio* transcript assembly and novel gene identification, Tiling Assembly, to compensate for such shortfalls.

By combining Tiling Assembly with Cufflinks,^10^ thousands of potential novel genes were identified in the rice genome. To reduce the possibility of false-positive novel gene identification, stringent filters on minimum gene length, minimum gene expression level, and percent similarity of the potential novel genes to another region in the genome were included. Utilizing this pipeline, 767 high-confidence, unannotated genes were identified in the rice genome. By applying Tiling Assembly to other model organisms, we identified 200 potential novel genes in *Arabidopsis thaliana*, 126 in *Caenorhabditis elegans*, 361 in *Drosophila melanogaster*, and 460 in *Saccharomyces cerevisiae*. This study demonstrated that, by utilizing Tiling Assembly, many potential novel genes can be identified in even the most well-annotated genomes.

## 2. Materials and methods

### 2.1. The Tiling Assembly pipeline

**Figure 1.**
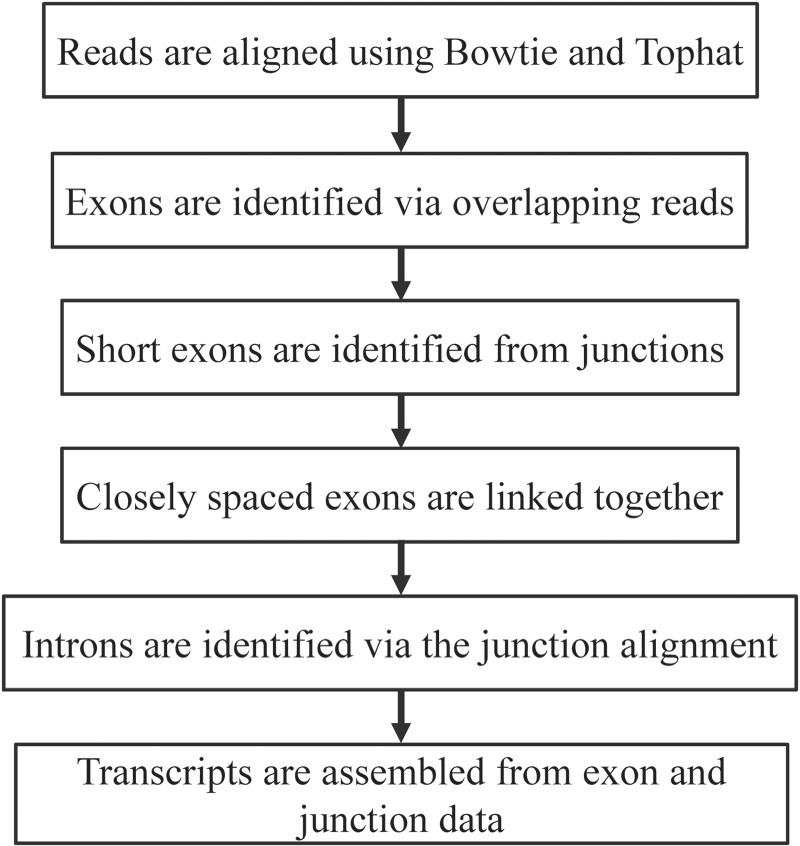
Flowchart of the processes for gene identification by Tiling Assembly.

The Tiling Assembly pipeline, depicted in Fig. 1, begins with alignment of RNA-seq short-read data to the appropriate genome using the alignment software, Bowtie^10^ and Tophat.^11^ Exons of potential genes are identified based on the presence of regions containing overlapping reads. Exons too short to be identified by read alignment are identified by Tophat junction alignments. Gaps in the read coverage can cause single exons to be identified as two or more exons; thus, exons that are very closely spaced are linked together. To prevent accidental merging of exons, exons containing one or more junction alignments are separated into multiple exons. Finally, all of the exons identified are joined together by the junction alignments to form the assembled transcripts.

Gene identification begins with the detection of exons based on the short-read data. The aligned reads are loaded into a MySQL database, which is queried by Tiling Assembly to identify exons based on the presence of overlapping reads. To prevent misidentification of exons due to noise, a threshold may be set by the user to determine the minimum reads per kilobase of exon (RPKE) required to identifying an exon.

Exons shorter than the length of a read (50 nt) tend to have few to no reads aligned, regardless of gene expression, preventing them from being detected by considering only read alignment. To identify these short exons, Tiling Assembly relies on the partial read alignments from junction mapping. Tophat takes the reads that cannot be directly mapped to the genome and breaks them into two parts for independent alignment. The junctions that these reads map across are indicative of an intron. Tiling Assembly relies on these partial read alignments to detect short exons (Supplementary Fig. S1).

Low read coverage from genes with low expression often leads to gaps in the read coverage, causing alignment algorithms to mistakenly identify multiple exons where only a single exon is present. To avoid such false gaps, Tiling Assembly merges exons that are within a user-specified distance of each other. Linking closely spaced exons together, however, may result in an incorrect merging of exons.

Other factors, such as intron retention, pre-spliced mRNA, and noise, may also contribute to incorrectly merged exons because they result in reads mapping to intronic regions (Supplementary Fig. S2). To prevent mistakenly merged exons, Tiling Assembly searches for junction alignments within the identified exons. Exons containing both sides of a junction alignment are separated and trimmed based on the boundaries of the junction to ensure accurate exon–intron boundaries (Supplementary Fig. S3). The beginning of the first exon and the end of the last exon of a transcript cannot be determined by junction alignment.

Once these high-confidence exons are produced, Tiling Assembly assembles the exons into specific genes using junction alignments. To avoid false junctions between similar genomic regions, the user can specify a maximum length of a junction that skips over one or more exons (Supplementary Fig. S4). In addition, the user can specify the size of very large junctions to be disregarded to avoid invalid junctions due to mapping errors.

### 2.2. RNA-seq and genome data

The RNA-seq data used for rice were obtained from RNA extraction of rice aleurone performed in our laboratory, followed by library preparation and sequencing on the Illumina Hi-seq 2000 platform by the Huntsman Cancer Institute, University of Utah. The data were submitted to the Sequence Read Archive (SRA)^12^ and are publicly accessible under the accession number SRP028376. The SRA accession numbers that were used for the analysis of other species were SRP022162 (*A. thaliana*), SRR590802-4 (*D. melanogaster*), SRR650494-5 (*C. elegans*), and SRR1019759 (*S. cerevisiae*).

The rice genome and annotation were downloaded from the MSU Rice Genome Annotation Project Release 7.0 (MSU R7)^13^ for *Oryza sativa* (ftp://ftp.plantbiology.msu.edu/pub/data/Eukaryotic_Projects/o_sativa/annotation_dbs/pseudomolecules/version_7.0/).
The *A. thaliana* data were downloaded from the PhytozomeV10^14^ (http://genome.jgi.doe.gov/pages/dynamicOrganismDownload.jsf?organism=PhytozomeV10).
The *C. elegans* data were downloaded from the WormBase^15^ (ftp://ftp.ensembl.org/pub/release-75/fasta/caenorhabditis_elegans/dna/).
The yeast data were downloaded from the *Saccharomyces* Genome Database^16^ (http://downloads.yeastgenome.org/sequence/S288C_reference/genome_releases/).
The *D. melanogaster* data were downloaded from the FlyBase^17^ (ftp://ftp.flybase.net/genomes/Drosophila_melanogaster/dmel_r5.56_FB2014_02/fasta/).

### 2.3. Alignment of full-length cDNA to the rice genome

The full-length cDNAs (FL-cDNAs)^18^ were aligned to the rice genome using the Exonerate alignment software with the following parameters: model est2genome, geneseed 250, and bestn 1.^19^ The FL-cDNAs and short-read data were loaded into the University of California, Santa Cruz (UCSC) Genome Browser,^20^ for visualization at the following address: http://shenlab.sols.unlv.edu/cgi-bin/hgGateway.

### 2.4. Short-read alignment

Short reads were aligned to MSU R7 via Bowtie version 2.1.0 and Tophat version 2.0.9 software and OLego.^21^ The maximum junction length was set to 50,000 nt. Default values were used for all other parameters. The short-read data for all of the samples were merged. Transcript assembly was then performed using Cufflinks version 2.0.2 on the composite data to generate GTF files. The mapped short reads and junctions were loaded into a MySQL database. Tiling Assembly queried the database to identify exons and genes.

### 2.5. Sample size calculation for comparison of Tiling Assembly and Cufflinks genes with FL-cDNA

The minimum sample size (*n*), needed to ensure the genes identified by Tiling Assembly and Cufflinks coincided with FL-cDNAs, was calculated using the following equation: $n=\frac{Nx}{\left[(N-1)E^{2}+x\right]}$, where $x={Z_{\left(\frac{c}{100}\right)}}^{2}r(100-r)$;^22^ *N* represents the total number of discrepant genes; *E* is the margin of error, which was set to 5%; the critical value $Z_{\left(\frac{c}{100}\right)}$ was set to 1.96 based on a 95% confidence level (*c*); and *r* was set to 60% since this value was the expected discrepancy rate contributed to alternative splicing.

## 3. Results and discussion

### 3.1. Short-read alignment

The short-read data from our previous publication were used in this study since we obtained the data and can vouch for its quality.^9^ In addition, using the same data allowed for comparison between our previously published results and the results in this study. The short reads were aligned to MSU R7 using the latest available version of Bowtie and Tophat. Of the 157,773,782 reads, 151,492,182 reads (96.0%) were aligned to the rice genome. Of the reads that aligned, over 10% aligned to currently unannotated regions, indicating that there are potentially many unidentified novel genes in the rice genome, similar to what was found for the human genome.^4^

Though the focus on this study is on transcript assembly rather than mapping, we compared Tophat with the splice-sensitive mapping tool OLego. Both programmes identified junctions that the other did not, indicating that neither programme was 100% accurate (Supplementary Fig. S5). Of the 158,314 junctions identified by OLego, 124,594 junctions (78.7%) matched identically with a junction identified by Tophat. Although OLego identified more junctions, most of the junctions uniquely identified by OLego (71.3%) were determined from a single read. Since nearly 80% of the OLego junctions matched identically with Tophat and most of the remaining were derived from a single read, we chose to keep within the Tuxedo suite software since Cufflinks was designed to work with Bowtie and Tophat. OLego may be used as an alternative mapping tool if so desired.

### 3.2. Benchmark analysis

**Figure 2.**
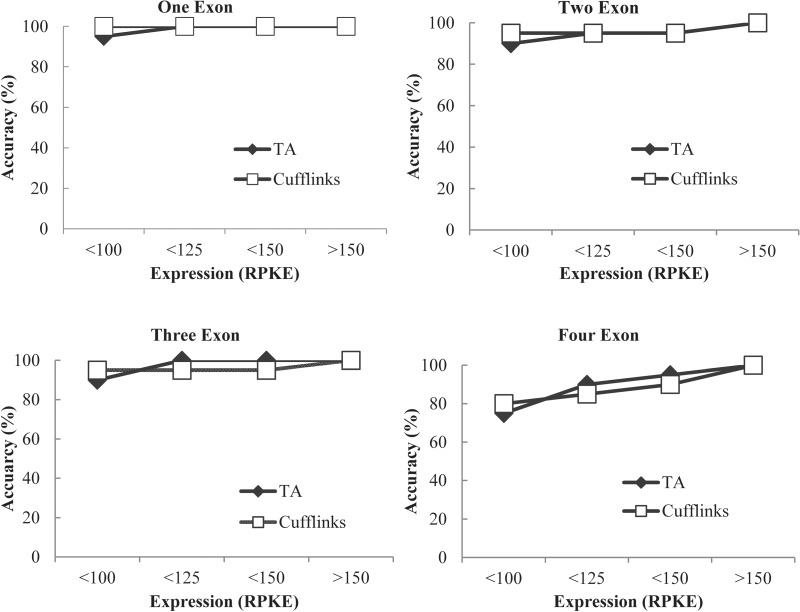
Tiling Assembly and Cufflinks show similar accuracy predicting known genes inserted in a random test genome. A random test genome containing 80 highly expressed, single-isoform genes was created. Four categories were used, containing 20 genes each: (A) one exon, (B) two exons, (C) three exons, and (D) four exons. The number of reads aligned to each gene was varied to determine the read depth at which each programme failed to accurately predict the genes.

To determine the minimum level of read alignment required for reliable detection of transcripts, 80 highly expressed single-isoform genes with evenly distributed reads across the exons were inserted into a randomly generated test genome. A control sample containing about 40 million reads was aligned to the test genome and not a single read mapped. This demonstrated that the test genome was an ideal method for excluding noise. Single-isoform genes were used to simplify the problem of correctly identifying all exons, while also allowing us to accurately determine the baseline for exon detection. Four categories were used, containing 20 genes each: one exon, two exons, three exons, and four exons. Tophat was used to align RNA-seq data from the control sample to the genes. Prior to analysing its ability to identify exons at a specific expression level, Tiling Assembly was run on the data to ensure the genes were properly identified as single-isoform genes with the appropriate number of exons. The minimum expression level used by Tiling Assembly to detect exons for these experiments was set to 50 RPKE, based on our analysis in Section 3.3., which indicated that expression levels below this threshold led to the identification of noise reads as exons. To test the ability of Tiling Assembly to correctly identify the genes at specific expression levels, 1,000 reads were randomly selected from the pool of reads that aligned to each gene and run through Tiling Assembly. The number of randomly selected reads was incrementally reduced, and Tiling Assembly was run again. This process was repeated several times until 50 reads per gene were selected. To compare the performance of Tiling Assembly with a well-established assembly algorithm, the same procedure was performed using Cufflinks. The accuracy with which Tiling Assembly and Cufflinks identified the genes was determined using a point of first failure method, which assumed that the highest RPKE, where a gene was first misidentified, was the point where identification becomes unreliable. As can be seen in Fig. 2, at an expression level of 100 RPKE, both Tiling Assembly and Cufflinks were able to identify single-exon and multi-exon genes with an 85% or higher accuracy rate. At a read depth lower than 100 RPKE, the accuracy rate dropped dramatically for both Cufflinks and Tiling Assembly, though Cufflinks' accuracy declined at a slower rate. These data indicate that Cufflinks and Tiling Assembly can accurately identify gene transcripts at a read depth of 100 RPKE or greater.

Cufflinks uses a parsimony algorithm to identify transcripts,^23^ using the smallest number of transcripts to explain all the reads. Thus, despite possible gaps in the reads, if the reads can be explained by a single transcript, Cufflinks assumes a single transcript. This assumption allows Cufflinks to correctly identify single-exon genes down to as low as 50 RPKE. The accuracy rates reported from the test genome favour Cufflinks; however, RNA-seq data in an actual genome would contain noise reads. These stray reads can cause Cufflinks to mistakenly identify them as individual genes or exons. In contrast, the set 50 RPKE cutoff utilized by Tiling Assembly for these experiments prevents such noise reads from being classified as genes.

To determine the rate of false-positive gene identification, 100 genes were indiscriminately selected and inserted into another random test genome. Both Tiling Assembly and Cufflinks found at least one gene at each of the locations, however, both also erroneously identified extra genes. Cufflinks reported 125 genes, whereas Tiling Assembly identified 117 genes. Investigation of the 25 extra genes identified by Cufflinks revealed numerous causes for misidentification, including missing junctions, gaps, and false identification of genes in intronic regions (Supplementary Figs S6–S8). While Tiling Assembly also overestimated the number of genes for similar reasons, it did so at a lower rate than Cufflinks. The main cause of this difference was due to identification of genes in intronic regions by Cufflinks, whereas Tiling Assembly disregarded these as noise.

Investigation on the ability of Tiling Assembly and Cufflinks to correctly identify exons in random test genomes revealed a trade-off between the two methods. While Cufflinks was better able to accurately identify genes in the test genome at expression levels lower than 100 RPKE, both programmes showed similar accuracy above 100 RPKE. Cufflinks was more likely to mistakenly identify intronic regions containing noise reads as genes, whereas Tiling Assembly's more conservative approach to exon identification decreased the identification of these false genes. Actual genomes contain many noise reads aligning on intronic and intergenic regions, in which case Tiling Assembly would identify fewer false-positive genes.

### 3.3. Application of Tiling Assembly identified 40,491 genes expressed in rice aleurone cells

To determine the gene identification capabilities of Tiling Assembly in an actual genome, Tiling Assembly was applied to rice aleurone short-read data composited from four samples and aligned to MSU R7. Prior to application of Tiling Assembly to the rice genome, it was necessary to determine the minimum gene expression required for accurate detection of exons. This was determined through analysis of several genes, with *LOC_Os01g01010* used as a representative example. Tiling Assembly was run multiple times on these genes, with varied expression thresholds, to determine at what point the exons were accurately recognized. The threshold was incrementally reduced from 100 RPKE. At 50 RPKE, Tiling Assembly identified exons e3 and e4 of *LOC_Os01g01010* (Supplementary Fig. S9); however, lower expression thresholds resulted in identification of false exons due to noise read alignment. A threshold of 50 RPKE was thus used to ensure accurate exon identification.

Exons were identified from MSU R7 using several steps. First, identification of exons was achieved through analysis of overlapping reads aligning to the same region of the genome, with minimum expression of 50 RPKE required for these regions to be considered an exon. This step led to an initial identification of 207,908 potential exons.

Identification of exons via tiling of read alignments limits the size of the exons found to the length of a single read. Tiling Assembly gets around this limitation via analysis of junction alignments produced by Tophat, as described in Section 2.1. In this second step of exon identification, an additional 1,397 potential exons were identified, bringing the total number to 209,305 potential exons.

When a gene has low read coverage, there may be exons which contain gaps in read alignment. Since Tiling Assembly depends on overlapping reads to identify exons, such gaps lead to artificial fragmenting of exons. To avoid this issue, exons that were within 50 nt of each other, a space which could be closed by the length of a single read in our data set, were merged together. To determine whether this merging of exons was likely to result in erroneous merging of genes, the annotated genes in MSU R7 were investigated. It was found that only 0.36% of the non-overlapping annotated genes reside within 50 nt of one another, so linking exons 50 nt apart should not cause a significant misrepresentation of the number of genes obtained from the overall RNA-seq analysis. However, linking closely spaced exons together may result in false merging of exons of the same gene. Of the 209,305 potential exons found in our RNA-seq data, 50,895 fragments were within 50 nt of each other. After linking these closely spaced fragments together, there were 158,410 potential exons.

To fix any exons that may have been mistakenly merged, either by noise or linking of exons, Tiling Assembly utilized the junction alignments produced by Tophat. Tiling Assembly searched for exons that contained a Tophat junction to identify any exons that may have been mistakenly merged. Our goal in this analysis with Tiling Assembly was to find the most common isoform of a gene where intron retention is a possibility. Thus, if the density of reads aligning on the Tophat junction was <50%, compared with those aligned to the adjacent regions, then the junction was considered to be an intron (Supplementary Fig. S10). This splitting of exons increased the total number of potential exons to 185,445 exons.

Once the exons were identified, the Tophat junction alignments were used to join exons together to form transcripts. Occasionally, Tophat maps false junctions across large distances due to sequence similarities to the actual junction elsewhere in the genome. To avoid considering these false junctions, Tiling Assembly was set to disregard junctions that skipped exons and spanned distances greater than 50k nt. In addition, in our previous study,^9^ it was found that there were numerous areas of the genome where large numbers of reads mapped to small regions, often <140 nt in length, due to high sequence similarity to other highly expressed regions of the genome. Tiling Assembly was thus set to disregard potential genes that had <140 nt. After linking all of the exons together and removing these very small genes, 40,491 genes were identified, containing 136,164 exons. This number does not represent the entire rice genome because Tiling Assembly relies on transcriptome data for gene identification and not all genes are expected to be expressed in all tissues, such as aleurone.

**Figure 3.**
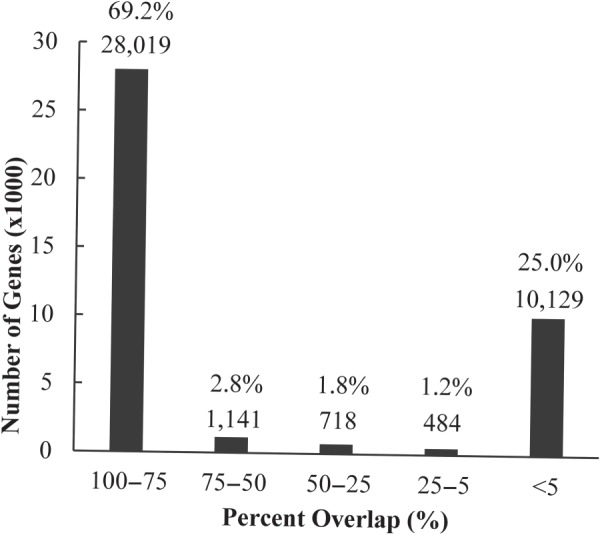
Genes identified by Tiling Assembly overlapped either well or minimally with annotated genes. The start and termination positions of genes identified by Tiling Assembly were compared with those of the MSU R7 genes to determine the amount of each Tiling Assembly gene corresponded with an MSU R7 gene. Of the 40,491 genes, 28,019 corresponded to an annotated gene by >75%, and 10,129 by <5%. This 5% category represents potential novel genes.

The genes found by Tiling Assembly were compared with the annotated genes in MSU R7. Of the 40,491 genes identified, 28,019 overlapped with an annotated gene by at least 75%, and 10,129 genes by <5% (Fig. 3). Thus, 94% of the genes identified by Tiling Assembly either corresponded well to an annotated gene or by a minimal amount. The 10,129 minimally overlapping genes were considered as potential novel genes.

The 2,343 Tiling Assembly genes that overlapped with an annotated gene between 5 and 75% may be the result of undiscovered alternatively spliced forms of known genes. Since these transcripts were identified using RNA-seq data from rice aleurone cells, a tissue that has not previously been used for gene identification, the presence of potential unannotated alternative splice variants of genes is not surprising.

### 3.4. Application of Cufflinks identified 38,175 genes expressed in rice aleurone cells

To compare the gene-finding capabilities of Tiling Assembly with a well-established assembly programme, Cufflinks was run on the same RNA-seq data. Of the 38,175 transcripts identified by Cufflinks, 32,969 overlapped with an MSU R7 annotation by at least 5%. The remaining 5,206 transcripts included multiple isoforms of the same genes. To reduce overrepresentation of the same gene, transcripts that were completely contained within another transcript were eliminated. This left 4,051 potential novel genes identified by Cufflinks. Of these, 48 genes were below the minimum 140 nt requirement used in this analysis and 18 genes were on unknown chromosomes. Therefore, 3,985 potential novel genes were identified by Cufflinks.

### 3.5. Comparison of the novel genes identified by Tiling Assembly and Cufflinks

**Figure 4.**
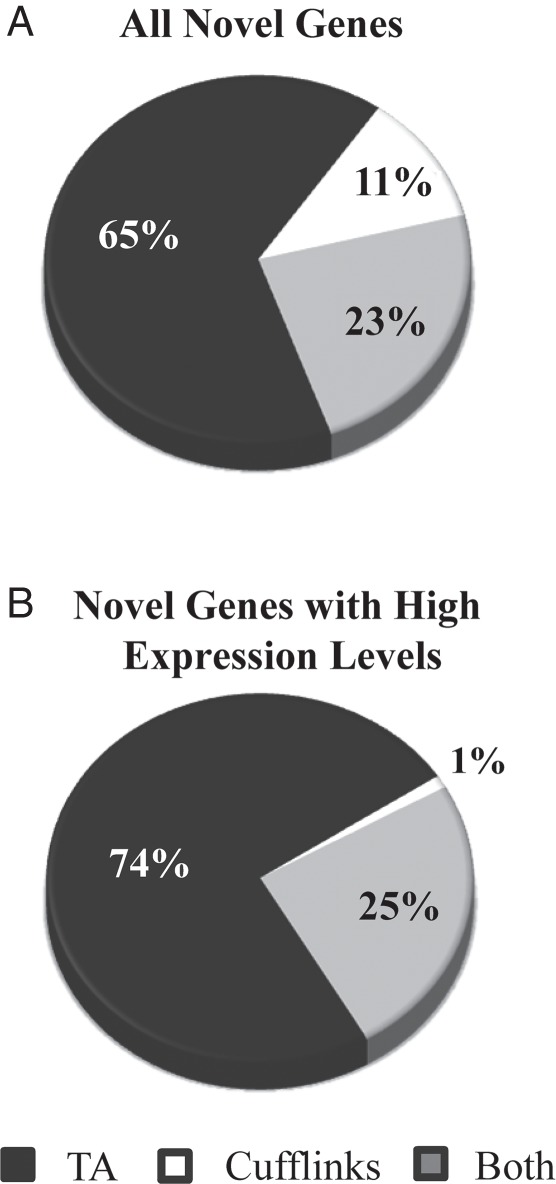
Tiling Assembly identified over 2.5 times more novel genes than Cufflinks. Novel genes identified by Tiling Assembly and Cufflinks were compared to determine how much they overlapped. (A) When all novel genes were considered, 7,460 genes were exclusively identified by Tiling Assembly, 1,316 by Cufflinks, and 2,669 by both. (B) When only novel genes with high expression levels (≥100 RPKE) were considered, 3,473 genes were exclusively identified by Tiling Assembly, 52 by Cufflinks, and 1,166 by both.

Among the potential novel genes identified by Tiling Assembly and Cufflinks, 1,316 genes were identified exclusively by Cufflinks, 7,460 by Tiling Assembly, and 2,669 by both (Fig. 4A). After eliminating potential novel genes with low expression, using 100 RPKE as a threshold for gene identification based on our benchmark analysis (Fig. 2), 4,690 genes were identified as potential novel genes. Of these, 3,473 genes were identified by Tiling Assembly, 52 by Cufflinks, and 1,166 by both (Fig. 4B).

### 3.6. BLAST searches were used to eliminate potential novel genes with high sequence similarity to other genomic regions, resulting in 767 high-confidence, unannotated novel genes

**Figure 5.**
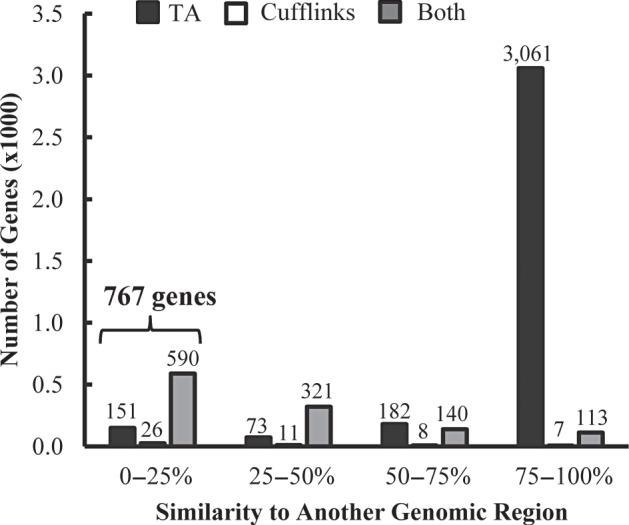
Tiling Assembly identified more novel genes than Cufflinks regardless of similarity to another genomic region. BLAST searches determined the level of similarity of the potential novel genes to other regions of the rice genome.

During read alignment, if a read can map to multiple locations within a genome, the read is randomly assigned to one of the locations.^1,24^ Because of this, potential novel genes that have a high similarity to another region in the rice genome may be false-positive genes. In addition, the rice genome contains a number of transcriptionally active gene fragments with high levels of sequence identity to annotated protein-coding genes and genes which may be involved in regulation of those genes rather than functioning as protein-coding genes themselves.^25^ BLAST^26^ searches revealed that 774 genes showed <25% sequence similarity to another region within the genome. Of these genes, seven genes had a footprint of <140 nt and were filtered out, bringing the total number of potential novel genes with <25% similarity to 767 genes (Fig. 5). These 767 genes were considered to be high-confidence novel genes based on the following criteria: they were unannotated, highly expressed, and contained relatively unique sequences (Supplementary Table S1). Of these high-confidence novel genes, 151 genes were uniquely identified by Tiling Assembly and 26 by Cufflinks. The remaining 590 genes were identified by both. Tiling Assembly was not only capable of finding 97% of the high-confidence novel genes found by Cufflinks, but it also found an additional 151 genes.

### 3.7. Comparison to previously published results

**Figure 6.**
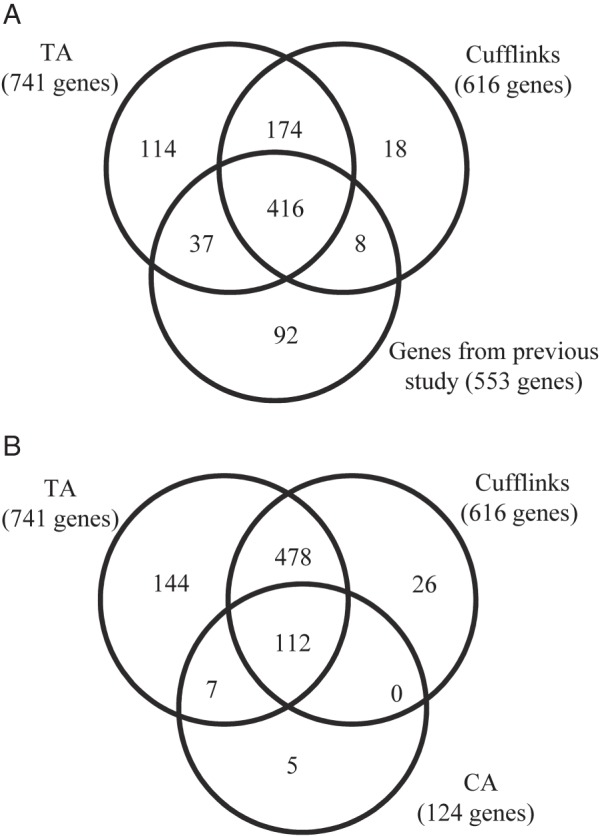
Comparison of Tiling Assembly and Cufflinks identified potential novel genes with those published in our previous study. (A) There were 92 genes identified in our previous study that were not classified as novel genes by Tiling Assembly or Cufflinks. While all of them were identified by Tiling Assembly or Cufflinks, slight changes in their length disqualified them from fitting into the category of potential novel genes. Of the 767 unannotated genes identified in this study, 306 genes were not identified in our previous study, demonstrating that our new pipeline is superior to that previously reported. (B) While all of the Clustering Algorithm genes were also found by Tiling Assembly, slight changes in their identification disqualified five from fitting into the category of potential novel genes.

In our previous publication, we identified 553 novel genes using a combination of Cufflinks and a custom Clustering Algorithm.^9^ Clustering Algorithm was developed to identify novel genes based on the presence of reads aligning to unannotated regions of the rice genome. Comparing the 767 potential novel genes identified by Tiling Assembly and Cufflinks with the 553 novel genes identified in our previous publication, there were 461 genes that coincided (Fig. 6A). There were 306 genes that were not identified in our previous publication. These additional 306 genes demonstrate that our new pipeline is superior to that previously reported.

There were 92 genes identified in our previous publication that were not considered as novel genes in this study. Most of these 92 genes were identified by Tiling Assembly in the initial steps but, due to slight differences in gene length, were filtered out as a result of low RPKE, high similarity to another region of the genome or overlap with an annotated gene. In addition, an older version of the rice genome and older versions of Cufflinks, Tophat, and Bowtie was used to identify genes in our previous publication. These factors resulted in a difference in the number of genes identified by Cufflinks in our previous publication, when compared with those reported in this study.

Of the 553 genes identified in our previous study, 124 were identified by Clustering Algorithm. These Clustering Algorithm genes were compared with Tiling Assembly and Cufflinks to determine the efficiencies of the algorithms (Fig. 6B). Of the Clustering Algorithm genes, 112 genes were found by both Tiling Assembly and Cufflinks. There were five Clustering Algorithm genes that were not identified by Tiling Assembly or Cufflinks.

Upon closer inspection of these five genes, it appeared Tiling Assembly correctly identified genes within the same location; however, there were differences in how the genes were identified. Two genes identified by Clustering Algorithm were associated with previously annotated genes by Tiling Assembly and were not considered novel genes. One Clustering Algorithm gene was identified as two genes by Tiling Assembly, each of which was eliminated as a potential novel gene based on similarity to another genomic region. Slight differences in the boundaries of the remaining two genes, as identified by Tiling Assembly, resulted in shorter genes. This resulted in an increased percent similarity to another genomic region, thus eliminating them as potential novel genes. Though these five genes may be potential novel genes, they do not satisfy our requirements for consideration as high-confidence potential novel genes.

### 3.8. Open reading frame identification

**Figure 7.**
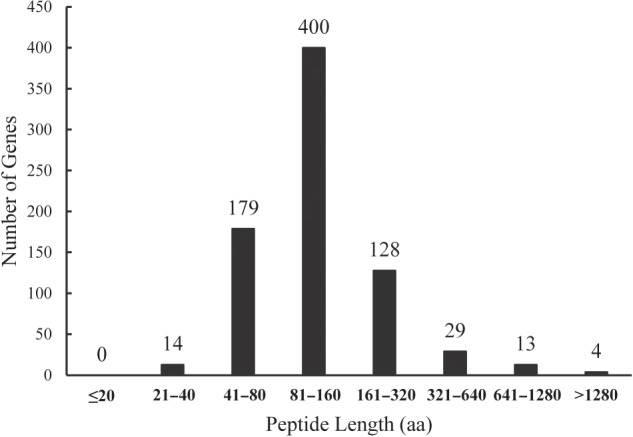
The peptide length distribution of potential novel genes is similar to that of annotated genes. The longest ORF was determined for each of the genes using an internally developed programme. The ORFs ranged from 23 to 4,737 codons in length, with an average length of 155 codons.

Many regions of the genome are actively transcribed, but do not produce protein products. To determine whether Tiling Assembly and Cufflinks identified novel protein-coding genes, the introns were removed and the longest possible open reading frame (ORF) associated with each of the 767 potential novel genes was determined. The predicted peptide lengths ranged from 23 to 4,737 codons. The average ORF length was 155 codons and more than half of the genes were 80–160 codons in length (Fig. 7). Since random DNA sequences are statistically unlikely to be more than 50 codons long without containing a stop codon,^27^ the fact that most of the ORFs found code for longer sequences indicates that they are likely protein-coding genes. Though proteins as small as 20 amino acids have been discovered in other organisms, they are uncommon,^28^ and no ORFs with fewer than 23 codons were found in our data set. Only 14 novel genes (1.8%) had predicted ORFs <40 codons and are probably not protein-coding genes. These genes may encode micro-RNAs or other non-coding RNAs.^28,29^ In addition, during the development of MSU R7, a 50 codon threshold was used. These data indicate that the majority of high-confidence novel genes identified by Tiling Assembly and Cufflinks are likely protein-coding genes, and it is not likely that they are genes that merely failed to meet the MSU R7 50 codon threshold.

The 767 potential novel genes identified by Tiling Assembly were further analysed by performing a protein BLAST on each gene to determine whether they exhibited any sequence homology to known proteins. There were 641 genes that showed some level of sequence homology, with 99 genes having an *E*-value ≤0.0001. Of these, 97 genes were homologous to predicted proteins, one to a hypothetical protein, and one to a bacterial heat-shock protein. The remaining 126 genes did not exhibit any sequence homology to known proteins. These genes may encode lincRNAs, or other long non-coding RNAs.^29^

### 3.9. Comparing Tiling Assembly and Cufflinks genes to FL-cDNAs

MSU R7 compiles annotation data from multiple sources, including FL-cDNA sequences, ESTs, and gene prediction software.^13^ As such, many of the annotated genes are hypothetical and not known to be expressed. To further evaluate the accuracy of Tiling Assembly in the identification of expressed genes, the genes identified by Tiling Assembly were compared with over 28,000 published FL-cDNAs, collected and sequenced by Kikuchi et al.^18^ These FL-cDNAs represent mRNA transcripts obtained from the rice plant and are thus more reliable than the computationally predicted transcripts in MSU R7. Of the 26,302 genes identified by Tiling Assembly with an expression level of at least 100 RPKE, 7,104 overlapped with a published FL-cDNA by >90% of their sequences. If multiple FL-cDNA variants overlapped with the same Tiling Assembly gene, the FL-cDNA variant with the same number of exons as the Tiling Assembly gene was selected for comparison. There were 5,767 genes that matched in exon number with their corresponding FL-cDNAs. The remaining 1,337 genes (18.8%) are here referred to as discrepant genes. To determine the source of these discrepancies between Tiling Assembly and the FL-cDNA, seven different categories of classification were used: extra exon, missing exon, extra intron, missing intron, missing junction, gap, or multiple discrepancies. In the instance Tiling Assembly recognized an exon where the corresponding FL-cDNA did not, the discrepancy was categorized as an extra exon (Supplementary Fig. S11A). In the instance Tiling Assembly did not identify an exon where the corresponding FL-cDNA did, it was categorized as a missing exon (Supplementary Fig. S11B). In the instance Tiling Assembly recognized an intron within the corresponding exon of the FL-cDNA, it was categorized as an extra intron (Supplementary Fig. S11C). In the instance Tiling Assembly recognized a single exon where the corresponding FL-cDNA recognized two exons, it was categorized to be a missing intron (Supplementary Fig. S11D).

The analysis was also performed on the Cufflinks genes. Of the 26,876 genes identified by Cufflinks that had an expression level of at least 100 RPKE, 7,690 overlapped with a published FL-cDNA by >90% of their sequences. Of these, 5,970 genes matched in exon number with their corresponding FL-cDNA and the remaining 1,720 genes (22.4%) were considered discrepant genes. Though Cufflinks identified more matching genes, the percentage and the number of discrepant genes were greater than Tiling Assembly.

**Figure 8.**
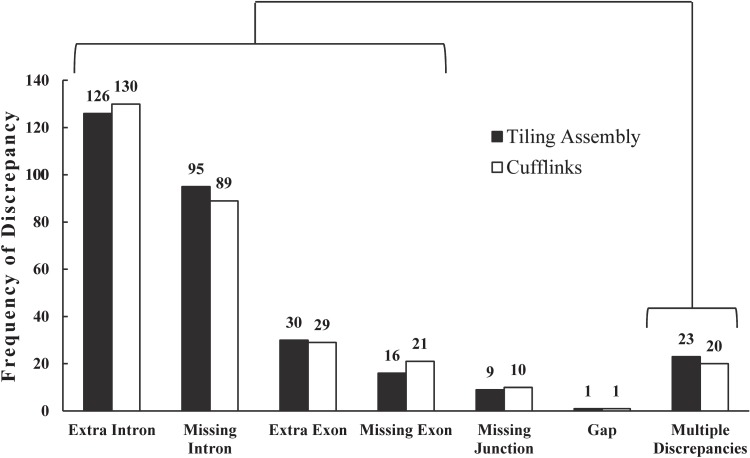
The major cause of discrepancies between Tiling Assembly and Cufflinks and their corresponding FL-cDNAs were due to extra or missing introns. A sample of 300 FL-cDNAs that had both corresponding Tiling Assembly and Cufflinks transcripts were analysed to determine the major causes of discrepancies. Extra intron and missing intron were the major causes of discrepancies for both Cufflinks and Tiling Assembly. Of the Tiling Assembly transcripts, 96.7% of the discrepancies may be due to alternative splicing that resulted in extra introns, missing introns, extra exons, missing exons, or multiple discrepancies (shown in brackets in the above image). Of the Cufflinks transcripts, 96.3% of the discrepancies may be due to alternative splicing.

Because it is time-consuming to analyse the causes of discrepancies for the 1,337 Tiling Assembly genes and the 1,720 Cufflinks genes, a portion of the gene pool was sampled for detailed analysis. The appropriate sample size for this comparison was calculated to be 290 genes, based on a 95% confidence level and a 5% margin of error (see Section 2.5.), and was rounded up to 300 genes. FL-cDNAs that had both a corresponding Tiling Assembly and Cufflinks gene that were discrepant were chosen for manual analysis. If a gene exhibited numerous reads, but showed a difference in the number of exons between the two data sets, alternative splicing was considered a possible cause. More than 60% of multi-exonic genes in plants are alternatively spliced,^30^ with intron retention being the most common form of alternative splicing.^31^ It is unlikely that the FL-cDNA dataset^18^ contains all alternative splice variants of transcripts. In addition, Tiling Assembly was designed to identify a single splice variant. Therefore, it was expected that the majority of the discrepancies may be due to alternative splicing. Indeed, of the 300 discrepant Tiling Assembly genes analysed, 96.7% could be attributed to alternative splicing. Extra or missing introns were the most abundant cause of the discrepancy as would be expected for plants. The remaining 3.3% were attributed to missing junctions or gaps (Fig. 8). Similar results were obtained for the Cufflinks dataset, with 96.3% due to possible alternative splicing and the remaining 3.7% attributed to missing junctions or gaps.

Applying the results from the sample of 300 discrepant Tiling Assembly genes, out of the 1,337 discrepant genes, it was expected that about 1,293 discrepancies (96.7%) may be due to alternative splicing events. The remaining 44 genes (3.3%) were expected to be the result of missing junctions, gaps, or other discrepancies. These 44 genes represented 0.6% of the 7,104 genes that overlapped with the FL-cDNAs. Therefore, it was expected that Tiling Assembly may be as high as 99.4% accurate in the identification of genes with at least 90% overlap.

Appling the results from the sample of 300 discrepant Cufflinks genes, out of the 1,720 discrepant genes, it was expected that 1,656 discrepancies were possible alternative splicing events. The remaining 64 genes were expected to be the result of missing junctions, gaps, or other discrepancies. These 64 genes represented 0.8% of the 7,690 genes that overlapped with the FL-cDNAs. Therefore, it was expected that Cufflinks may be as high as 99.2% accurate in the identification of genes with at least 90% overlap.

### 3.10. Identification of transcription start and termination sites by Tiling Assembly

**Figure 9.**
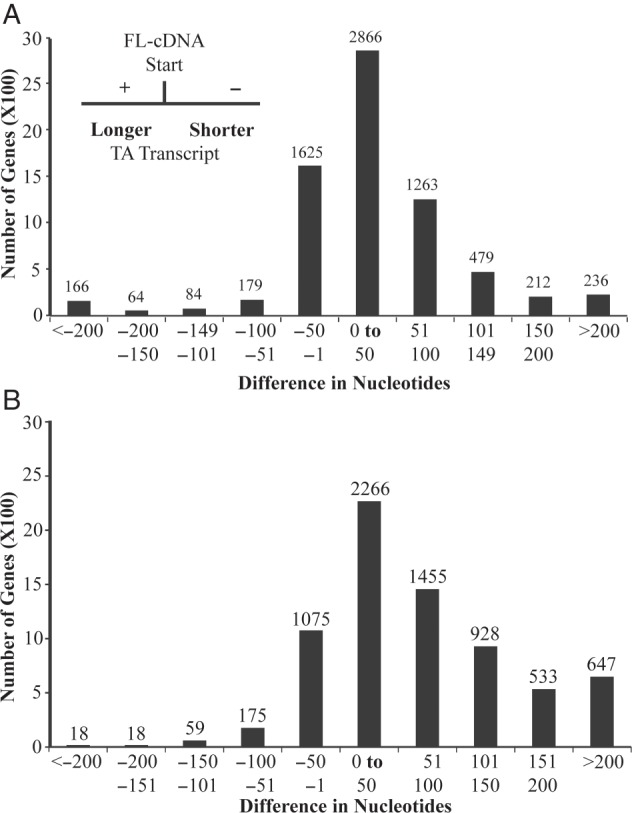
Nucleotide differences between transcriptional start (A) and termination (B) sites of FL-cDNAs and Tiling Assembly genes. The transcriptional start and termination sites were compared with those of FL-cDNAs previously published. The distribution of the difference between the Tiling Assembly and FL-cDNA start and stop sites is presented here. A negative value indicates that the Tiling Assembly gene is shorter than the FL-cDNA and a positive value indicates that the Tiling Assembly gene is longer.

The transcription start and termination sites were compared between genes identified by Tiling Assembly with those identified by the FL-cDNAs (Fig. 9). Only Tiling Assembly genes that overlapped with an FL-cDNA by at least 90% were considered. Of the 7,174 transcription start sites that satisfied the specified overlap threshold, ∼83% differed by ≤100 nt (Fig. 9A). The transcription start sites predicted by Tiling Assembly were on average 30 nt upstream of the FL-cDNAs. These data demonstrated that Tiling Assembly is a reasonably reliable tool for the identification of transcription start sites.

Of the 7,174 transcription termination sites that satisfied the specified overlap threshold, ∼69% differed by ≤100 nt (Fig. 9B). The transcription termination sites predicted by Tiling Assembly were on average 71 nt downstream of the FL-cDNAs. These data demonstrate that Tiling Assembly is less reliable at predicting the transcription termination sites. Overall, Tiling Assembly overestimated the length of the transcripts. This overestimation may be due to noise reads near the start and termination sites. Alternatively, past research has indicated that termination sites are variable.^32^ Hence, the accuracy rates of Tiling Assembly in predicting transcription termination sites may be underestimated.

### 3.11. Application of Tiling Assembly to the genomes of model organisms

Having demonstrated the effect of Tiling Assembly on detecting novel genes in rice, its performance was evaluated on other model organisms. Loraine et al.^33^ discovered 5,312 transcriptionally active regions (TARs) in the unannotated regions of the *A. thaliana* genome; however, few filters were used to remove false-positive TARs. For instance, of the 5,312 TARs reported, 3,490 were the length of a single read (75 nt). However, this large number of TARs indicated that there may still be undiscovered genes in Arabidopsis. Using the same Tiling Assembly parameters used for identifying potential novel genes in *O. sativa*, 218 potential novel genes were identified in Arabidopsis, representing nearly 1% of all annotated genes. Of these 218 potential novel genes, 99 genes (45%) contained at least part of one or more TARs, and 35 genes (16%) had at least one TAR completely contained within the gene. It is likely that most of the TARs reported by Lorraine et al. did not correlate with a Tiling Assembly novel gene because the majority of them were identified based on individual reads. These individual reads may have resulted from genomic DNA contamination or noise caused by statistical mapping error.

To determine whether the ability of Tiling Assembly to find large numbers of unannotated genes was applicable to non-plant species, the algorithm was applied to several additional model organisms (Table 1). Surprisingly, 458 novel genes were identified in *S. cerevisiae*, representing almost 7% of the known genes. Even though this model organism has been more intensively studied than Arabidopsis, this large number of potential novel genes may be due to the fact that only a single RNA-seq replicate was used in this study. Similar analysis was performed on *D. melanogaster* and *C. elegans*. The number of potential novel genes identified in each of these additional organisms may be improved by using parameters specific to the organism.

**Table 1.** Potential novel genes identified in other organisms by Tiling Assembly

Species	Ch	GS (Mb)	No. of genes	No. of novel genes	% Annot. genes
*O. sativa*	12	381	55,986	767	1.37
*A. thaliana*	5	125	25,498	218	0.85
*S. cerevisiae*	16	12	6,603	458	6.94
*C. elegans*	6	97	47,060	126	0.27
*D. melanogaster*	4	120	17,294	361	2.09

RNA-seq data downloaded from the SRA were used to investigate the ability of Tiling Assembly to find unannotated genes in additional model organisms. For each of the organisms, the same criteria were used for identifying potential novel genes as those used for rice.

Ch: number of chromosomes; GS: genome size; Annot. genes: annotated genes.

### 3.12. Discussion

There are relatively few publicly available transcript assembly programmes despite the vast increase in the use of RNA-seq. In this study, a novel assembly algorithm, Tiling Assembly, was developed to address the lack of established algorithms to identify transcribed regions as genes. This algorithm was compared with the Cufflinks transcript assembly software to evaluate its gene-finding capabilities in relation to established assembly software (Table 2). It was concluded that Tiling Assembly found substantially more genes and found a lower number of false-positive genes, though Cufflinks ran somewhat faster and was able to identify multiple transcripts for a given gene. It was also determined that Tiling Assembly and Cufflinks had similar accuracy at predicting single and multi-exonic genes down to 100 RPKE (Fig. 2). When the Tiling Assembly genes were compared to FL-cDNAs, the vast majority of discrepancies could be attributed to alternative splicing. Excluding those genes, Tiling Assembly appeared to be as high as 99.4% accurate in identification of genes.

**Table 2.** Comparison of Tiling Assembly to Cufflinks

Category	Tiling Assembly	Cufflinks
Novel gene finding^a^	3,473 genes	52 genes
False-positive rate^b^	17 out of 100 genes	25 out of 100 genes
Ease of use	User interface	Command line
Algorithm	Read tiling	Bipartite graph
Minimum expression	100 RPKE	100 RPKE
Run time^c^	3–4 h	1.5 h
Transcripts identified	Single transcript	Multiple transcripts

^a^These figures represent genes found exclusively by either TA or Cufflinks that have an expression level greater than 100 RPKE, prior to percent similarity filter.

^b^Data from random genome with 100 known genes inserted. No filtering performed.

^c^Excludes set-up time. Set-up for Tiling Assembly and Cufflinks is about the same time.

Before applying filters for potential novel genes, 28,019 genes that overlapped at least 75% with an annotated gene (Fig. 3) were found from the RNA-seq data using Tiling Assembly. MSU R7 annotation contains 55,986 genes, which would seem to imply that half the annotated genes in the rice genome were expressed in the aleurone cells. In our previous publication, we reported that 18,152 annotated genes were expressed in the aleurone cells.^9^ The criteria used for expression in our previous publication required an expression level of at least 1.0 read per kilobase of exon model per million mapped reads (RPKM), which is equivalent to ∼150 RPKE for this data. At 150 RPKE, Tiling Assembly identified 17,971 annotated genes, which were in line with our previous publication. Since 100 RPKE was determined as the minimum expression for gene detection, Tiling Assembly identified 20,230 expressed annotated genes in rice aleurone.

Comparison of the novel genes identified by Tiling Assembly with Cufflinks shows that Tiling Assembly identifies up to 74% more genes than Cufflinks (Fig. 4). After eliminating genes that showed high similarity to another genomic region, the Tiling Assembly identified 151 more novel genes than Cufflinks (Fig. 5). We analysed the data to determine possible causes for the large discrepancy in genes identified between the two programmes. One possible reason for this discrepancy was that Tiling Assembly was calling regions that were highly similar to another region, whereas Cufflinks disregarded them. BLAST search filters revealed that this was not the case. Another possible reason was that Cufflinks and Tiling Assembly found a gene within the same region, but the start and stop locations of those genes were different. Investigation of the positional overlap of the high-confidence novel genes found by each of the programmes verified that those genes unique to each programme did not overlap. A final possibility considered was that some novel genes detected by Cufflinks were longer than the corresponding Tiling Assembly gene and thus overlapped with an adjacent annotated gene. This overlap eliminated the Cufflinks gene as a potential novel gene. Analysis showed that there were 101 Cufflinks genes that overlapped with an annotated gene. These occurrences were attributed to two causes; the Cufflinks gene included regions with low read depth, which were considered noise reads by Tiling Assembly, or Cufflinks exons were merged based on junction alignments that were disregarded by Tiling Assembly because they were very long and skipped exons. In five cases, one of these long junctions skipped over an expressed region, which was called a novel gene by Tiling Assembly but an intron by Cufflinks. In 19 additional cases, these long junction alignments led to extremely long genes identified by Cufflinks which spanned multiple MSU R7 annotated and Tiling Assembly genes. There were nine expressed regions detected as a gene by Tiling Assembly where there was no corresponding Cufflinks gene, for unknown reasons.

Tiling Assembly is a heuristic, *ab initio* transcript assembly algorithm, which uses a read tiling approach to identify transcripts. Unlike *de novo* assembly algorithms such as Trinity,^5^ Tiling Assembly takes advantage of a sequenced genome to improve the accuracy of transcript assembly while decreasing CPU requirements. Tiling Assembly does not require an annotated genome, so it may be used for organisms where the genome is sequenced but the annotation is naive. Many of the current transcript assembly algorithms attempt to reproduce each of the isoforms available to a gene using a bipartite graph approach, which can lead to reporting of statistically probable, but non-real isoforms and dilution of expression levels of real isoforms as reads are assigned to the non-real isoforms. Tiling Assembly instead produces the longest possible isoform of a gene. Comparison of Tiling Assembly with a well-established transcript assembly programme, Cufflinks, revealed that Tiling Assembly's strengths lie in the accurate prediction of exons in the presence of noise and improved discovery of high-confidence novel genes.

In conclusion, we describe a heuristic approach to novel gene identification. Using this approach in combination with Cufflinks, 767 high-confidence unannotated genes were identified in rice. These genes contained predicted ORFs ranging from 40 to over 4,000 codons, with the majority showing sequence homology to known and predicted proteins. The accuracy of the genes identified by Tiling Assembly was validated through comparison with their corresponding FL-cDNAs, which implied Tiling Assembly may be as accurate as 99.4%. Tiling Assembly accurately predicted the transcription start sites to within 100 nt of the corresponding FL-cDNA, but was less accurate at predicting the transcription termination sites. Application of Tiling Assembly on *A. thaliana*, *D. melanogaster*, *C. elegans*, and *S. cerevisiae* identified hundreds of high-confidence novel genes, demonstrating that even in the most well-studied model organisms there are still undiscovered genes. This pipeline proves to be an effective way to identify novel genes in a diverse array of organisms. The novel genes identified here should be further studied to determine their functions and roles in their organisms.

## 4. Availability of software

Project name: Tiling Assembly

Project home page: http://shenlab.sols.unlv.edu/shenlab/

Operating systems: Platform independent

Programming language: PERL

Other requirements: MySQL or SQLite

License: Open Source license GNU General Public License version 2.0

Restrictions to use by non-academics: license needed

## Supplementary data

Supplementary data are available at www.dnaresearch.oxfordjournals.org.

## Funding

This work is supported by a USDA grant (2008-35100-04519). Funding to pay the Open Access publication charges for this article was provided by University of Nevada, Las Vegas, USA.

## Supplementary Material

Supplementary Data
