# Supplementary material for: Tiling Assembly: a new tool for reference annotation-independent transcript assembly and novel gene identification by RNA-sequencing
Source: DNA Res. 2015 Sep 3;22(5):319–29. doi: 10.1093/dnares/dsv015 (PMC4596398; doi:10.1093/dnares/dsv015)
Supplement: Supplementary Data [file supp_22_5_319__index.html]

Tiling Assembly: a new tool for reference annotation-independent transcript assembly and novel gene identification by RNA-sequencing — Tiling Assembly: a new tool for reference annotation-independent transcript assembly and novel gene identification by RNA-sequencing — Supplementary Data 

# Tiling Assembly: a new tool for reference annotation-independent transcript assembly and novel gene identification by RNA-sequencing

## Supplementary Data

Supplementary Data

- Supplementary Figures - pptx file
- Supplementary Table 1 - xlsx file
