# Supplementary material for: Tiling Assembly: a new tool for reference annotation-independent transcript assembly and novel gene identification by RNA-sequencing
Source: DNA Res. 2015 Sep 3;22(5):319–29. doi: 10.1093/dnares/dsv015 (PMC4596398; doi:10.1093/dnares/dsv015)
Supplement: Supplementary Data [file supp_dsv015_dsv015supp_figures.pptx]

## Slide 1
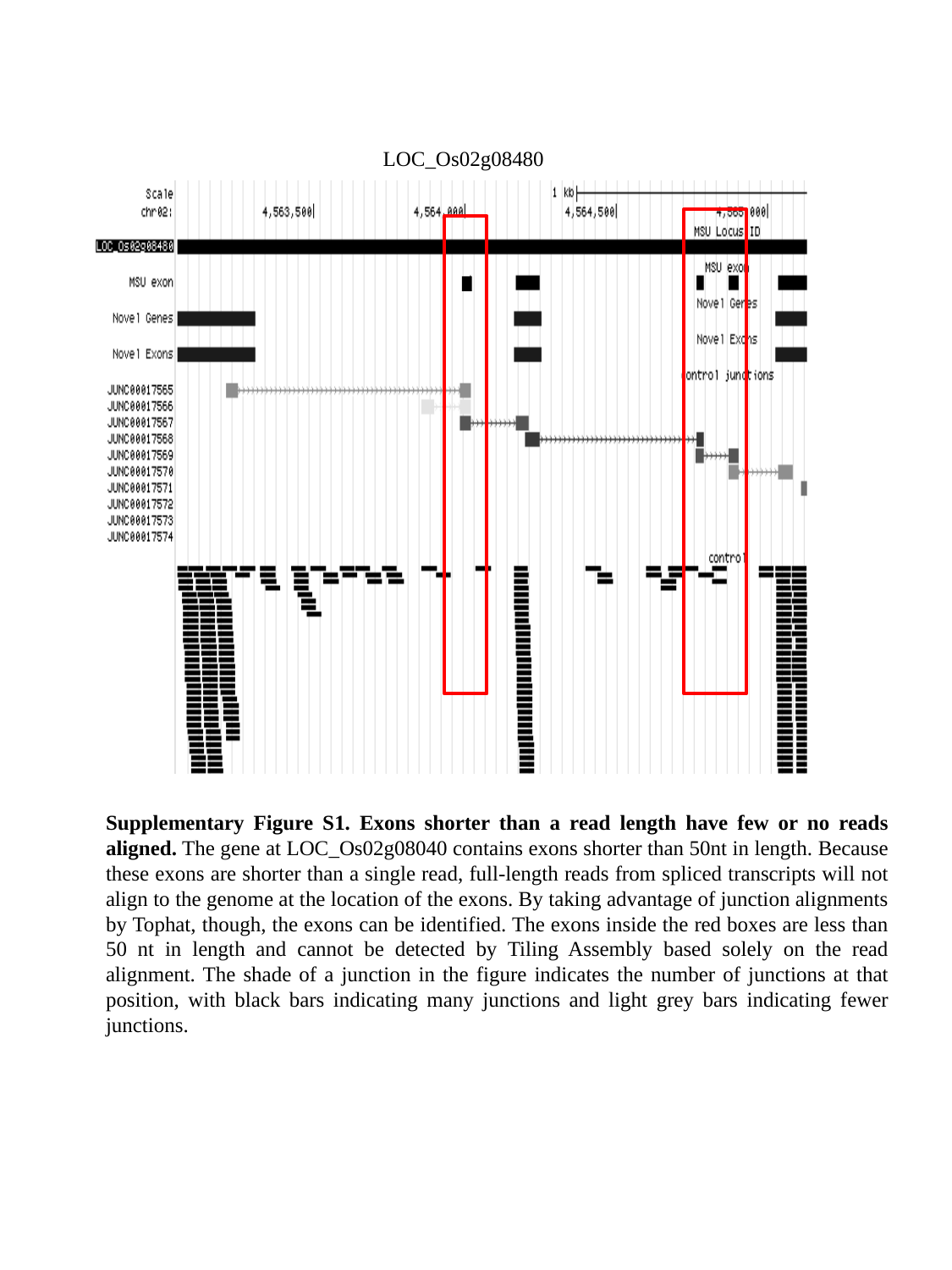

LOC_Os02g08480
Supplementary Figure S1. Exons shorter than a read length have few or no reads aligned. The gene at LOC_Os02g08040 contains exons shorter than 50nt in length. Because these exons are shorter than a single read, full-length reads from spliced transcripts will not align to the genome at the location of the exons. By taking advantage of junction alignments by Tophat, though, the exons can be identified. The exons inside the red boxes are less than 50 nt in length and cannot be detected by Tiling Assembly based solely on the read alignment. The shade of a junction in the figure indicates the number of junctions at that position, with black bars indicating many junctions and light grey bars indicating fewer junctions.

## Slide 2
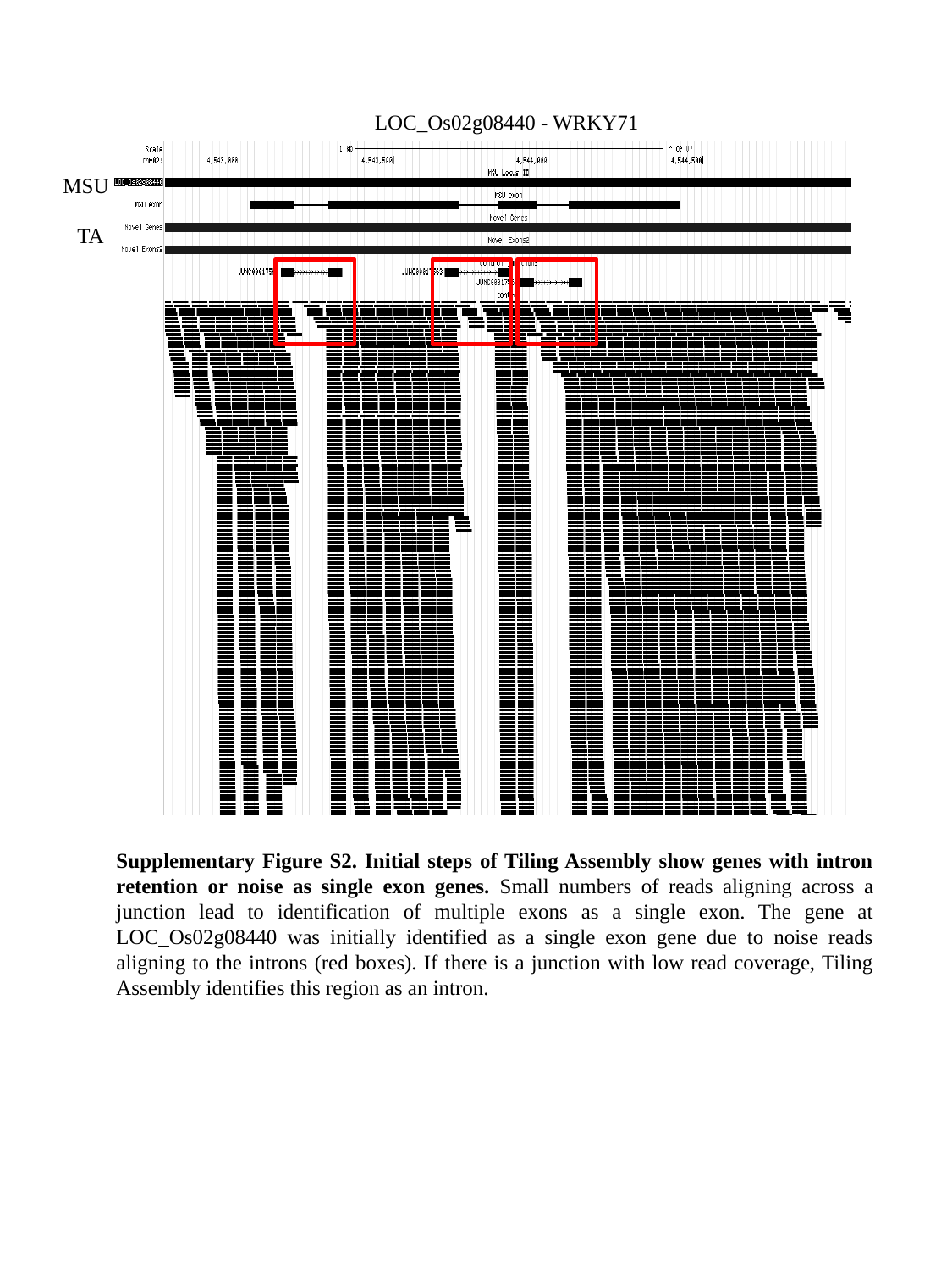

LOC_Os02g08440 - WRKY71
MSU
TA
Supplementary Figure S2. Initial steps of Tiling Assembly show genes with intron retention or noise as single exon genes. Small numbers of reads aligning across a junction lead to identification of multiple exons as a single exon. The gene at LOC_Os02g08440 was initially identified as a single exon gene due to noise reads aligning to the introns (red boxes). If there is a junction with low read coverage, Tiling Assembly identifies this region as an intron.

## Slide 3
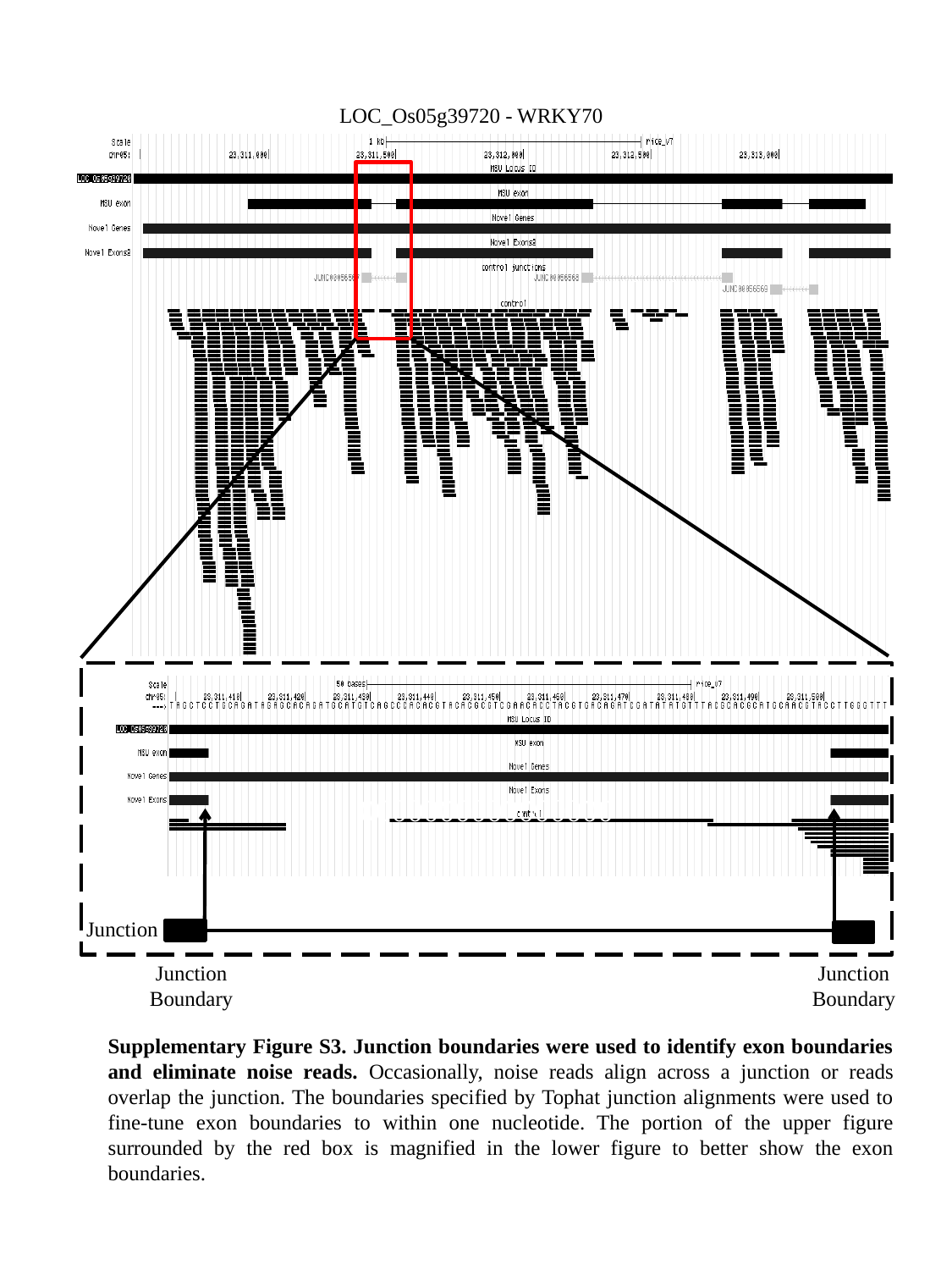

LOC_Os05g39720 - WRKY70
0000000000000000
Junction
Junction Boundary
Junction Boundary
Supplementary Figure S3. Junction boundaries were used to identify exon boundaries and eliminate noise reads. Occasionally, noise reads align across a junction or reads overlap the junction. The boundaries specified by Tophat junction alignments were used to fine-tune exon boundaries to within one nucleotide. The portion of the upper figure surrounded by the red box is magnified in the lower figure to better show the exon boundaries.

## Slide 4
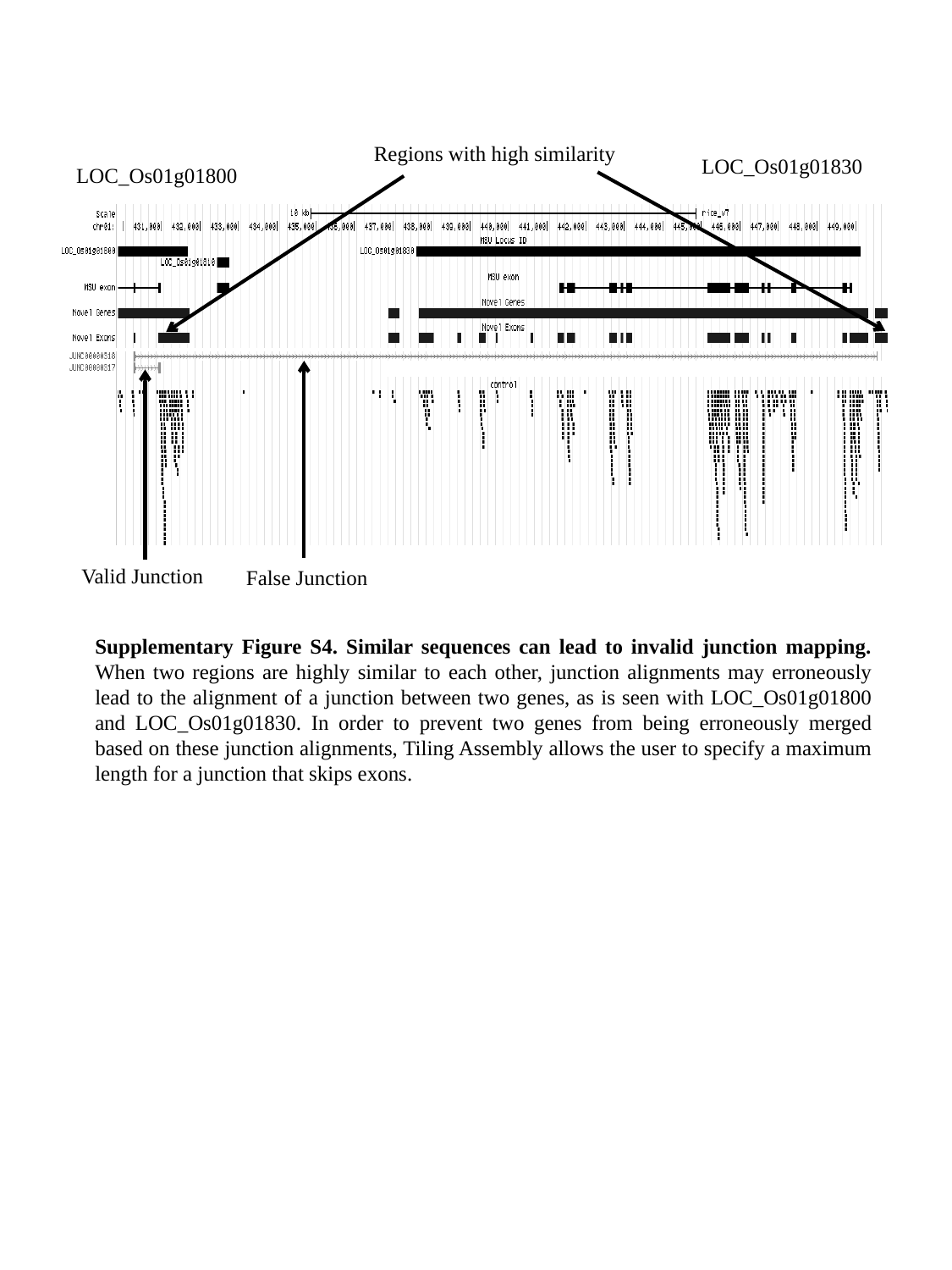

Regions with high similarity
LOC_Os01g01830
LOC_Os01g01800
Valid Junction
False Junction
Supplementary Figure S4. Similar sequences can lead to invalid junction mapping. When two regions are highly similar to each other, junction alignments may erroneously lead to the alignment of a junction between two genes, as is seen with LOC_Os01g01800 and LOC_Os01g01830. In order to prevent two genes from being erroneously merged based on these junction alignments, Tiling Assembly allows the user to specify a maximum length for a junction that skips exons.

## Slide 5
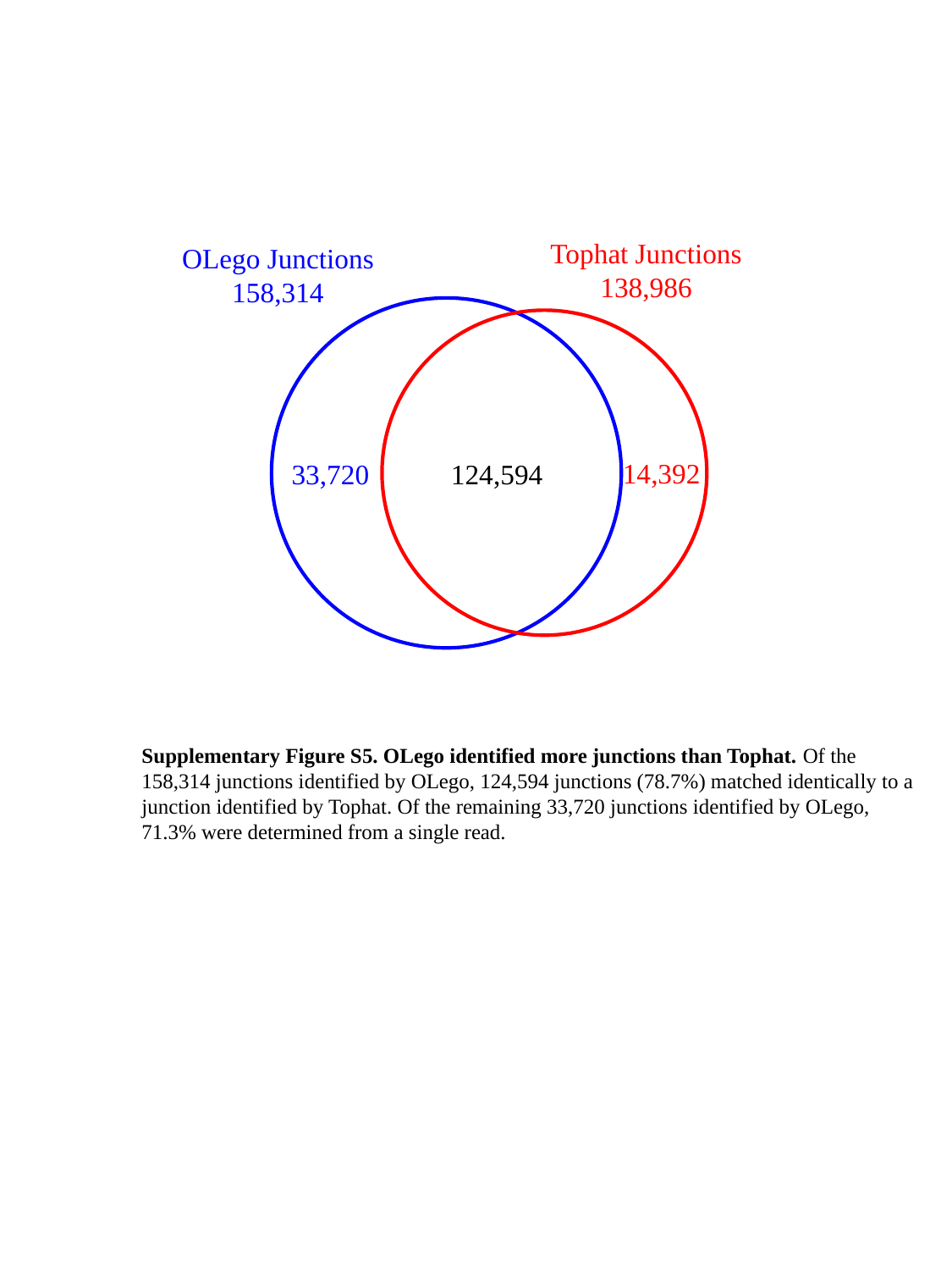

Tophat Junctions
138,986
OLego Junctions
158,314
14,392
33,720
124,594
Supplementary Figure S5. OLego identified more junctions than Tophat. Of the 158,314 junctions identified by OLego, 124,594 junctions (78.7%) matched identically to a junction identified by Tophat. Of the remaining 33,720 junctions identified by OLego, 71.3% were determined from a single read.

## Slide 6
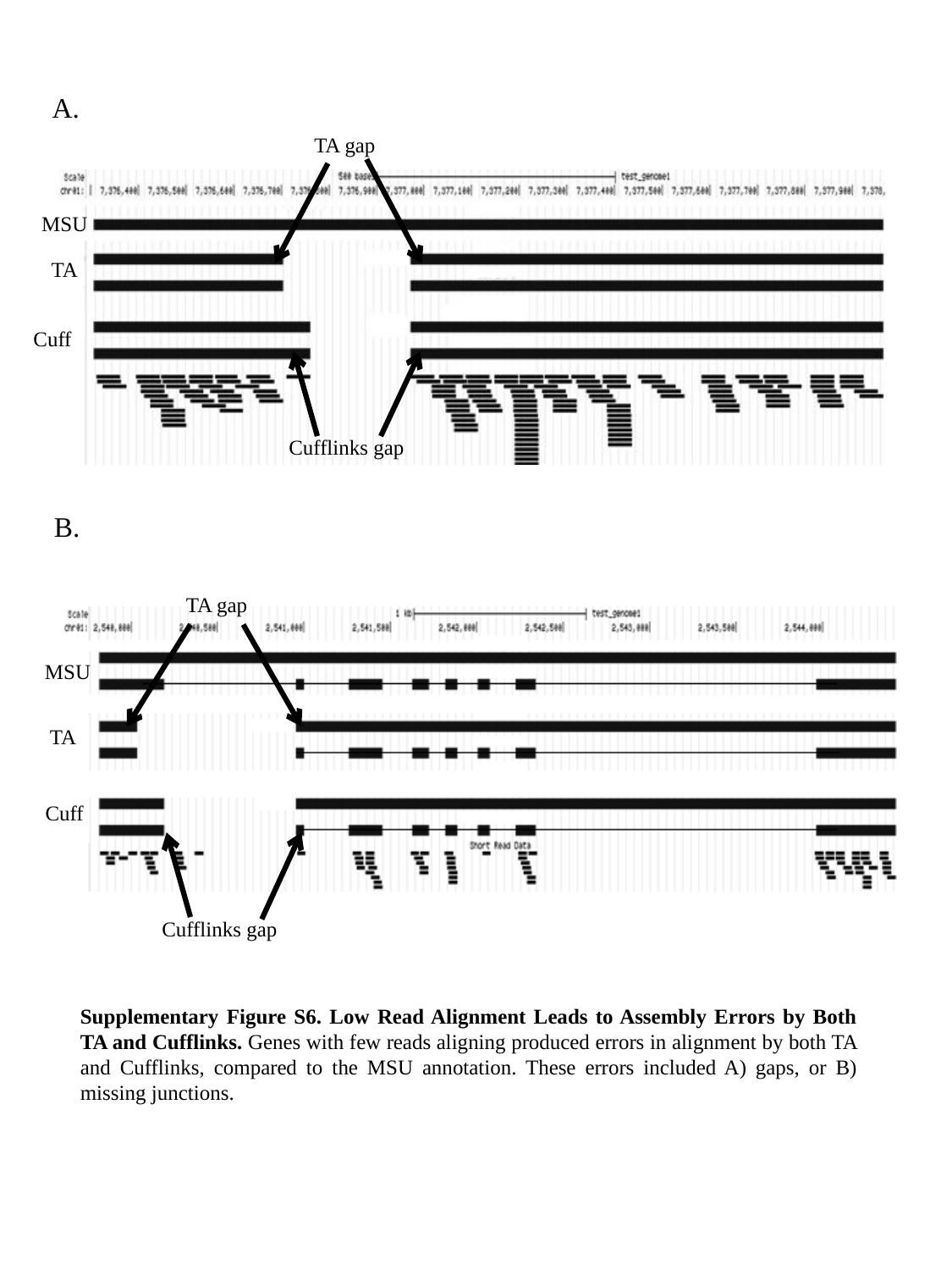

A.
TA gap
MSU
TA
Cuff
Cufflinks gap
B.
TA gap
MSU
TA
Cuff
Cufflinks gap
Supplementary Figure S6. Low Read Alignment Leads to Assembly Errors by Both TA and Cufflinks. Genes with few reads aligning produced errors in alignment by both TA and Cufflinks, compared to the MSU annotation. These errors included A) gaps, or B) missing junctions.

## Slide 7
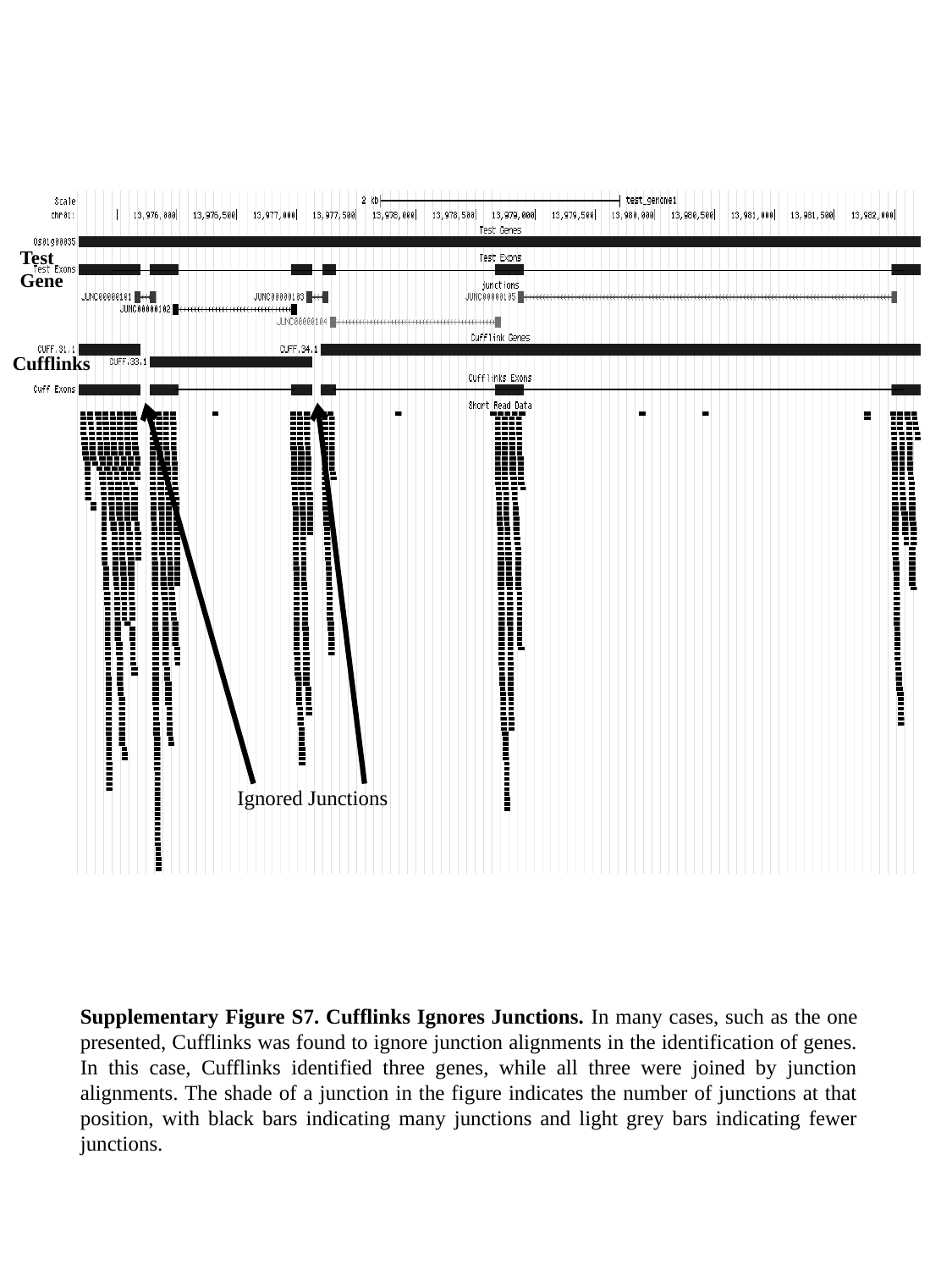

Test Gene
Cufflinks
Ignored Junctions
Supplementary Figure S7. Cufflinks Ignores Junctions. In many cases, such as the one presented, Cufflinks was found to ignore junction alignments in the identification of genes. In this case, Cufflinks identified three genes, while all three were joined by junction alignments. The shade of a junction in the figure indicates the number of junctions at that position, with black bars indicating many junctions and light grey bars indicating fewer junctions.

## Slide 8
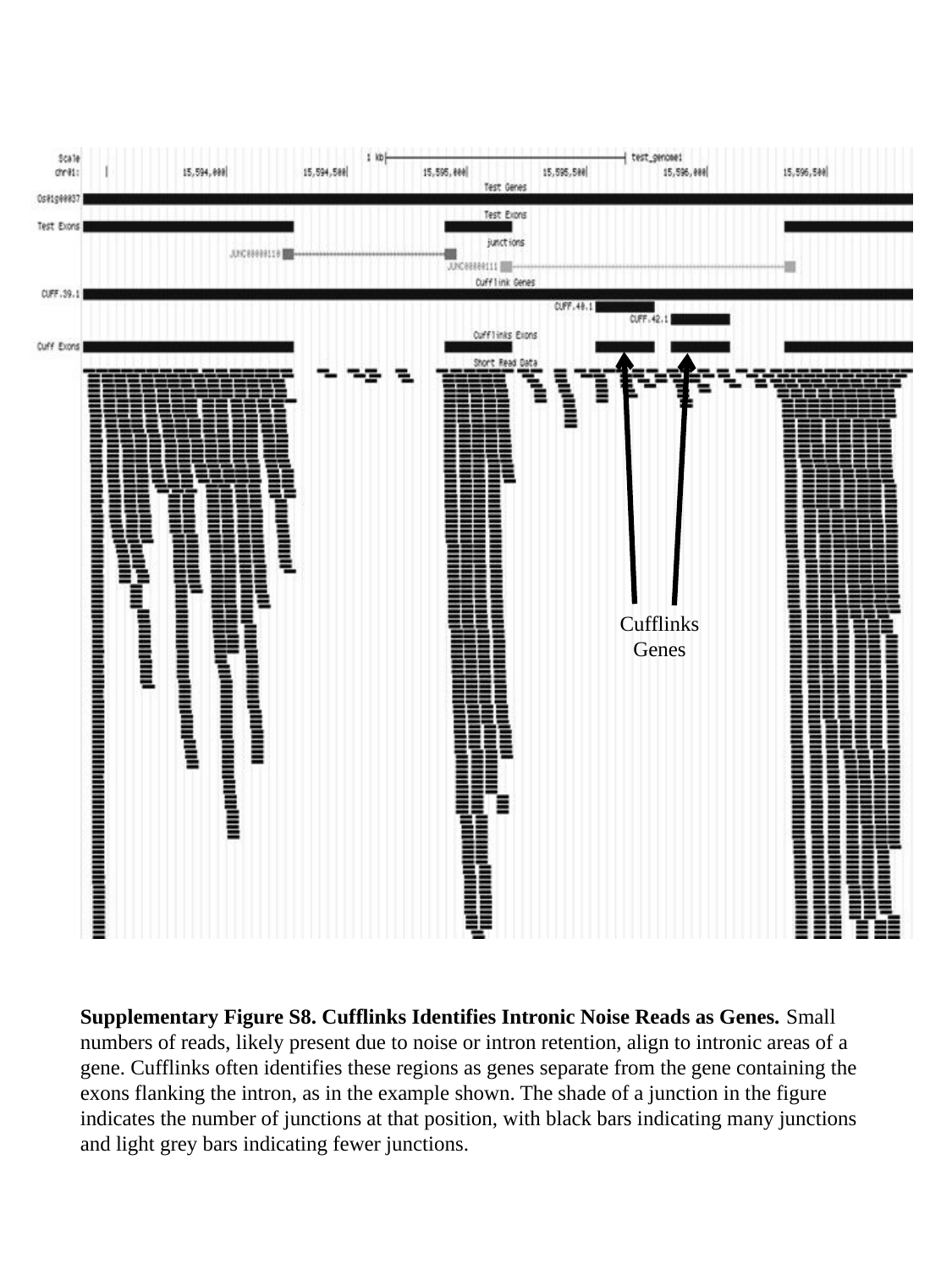

Cufflinks Genes
Supplementary Figure S8. Cufflinks Identifies Intronic Noise Reads as Genes. Small numbers of reads, likely present due to noise or intron retention, align to intronic areas of a gene. Cufflinks often identifies these regions as genes separate from the gene containing the exons flanking the intron, as in the example shown. The shade of a junction in the figure indicates the number of junctions at that position, with black bars indicating many junctions and light grey bars indicating fewer junctions.

## Slide 9
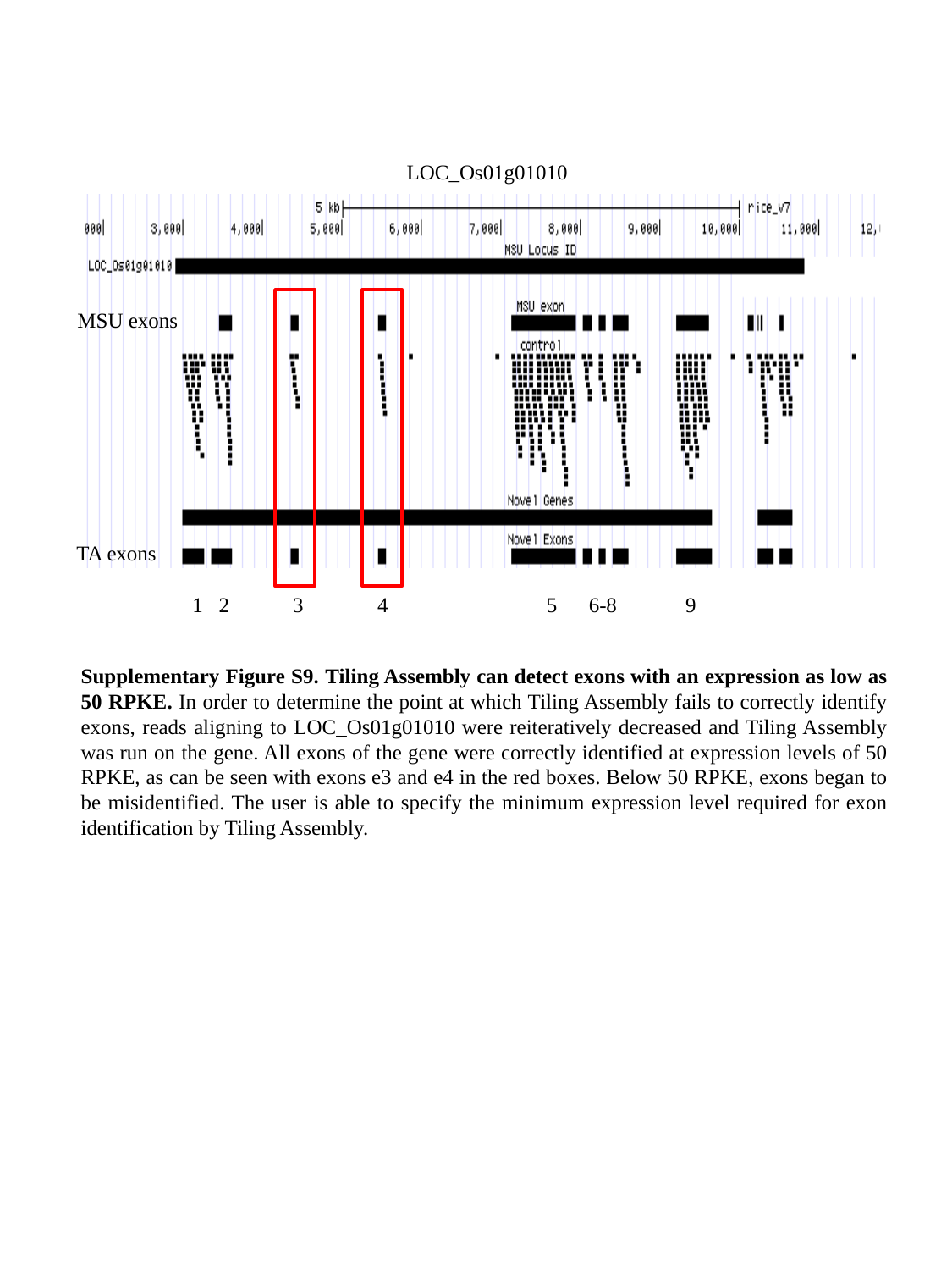

LOC_Os01g01010
MSU exons
TA exons
 1 2 3 4 5 6-8 9
Supplementary Figure S9. Tiling Assembly can detect exons with an expression as low as 50 RPKE. In order to determine the point at which Tiling Assembly fails to correctly identify exons, reads aligning to LOC_Os01g01010 were reiteratively decreased and Tiling Assembly was run on the gene. All exons of the gene were correctly identified at expression levels of 50 RPKE, as can be seen with exons e3 and e4 in the red boxes. Below 50 RPKE, exons began to be misidentified. The user is able to specify the minimum expression level required for exon identification by Tiling Assembly.

## Slide 10
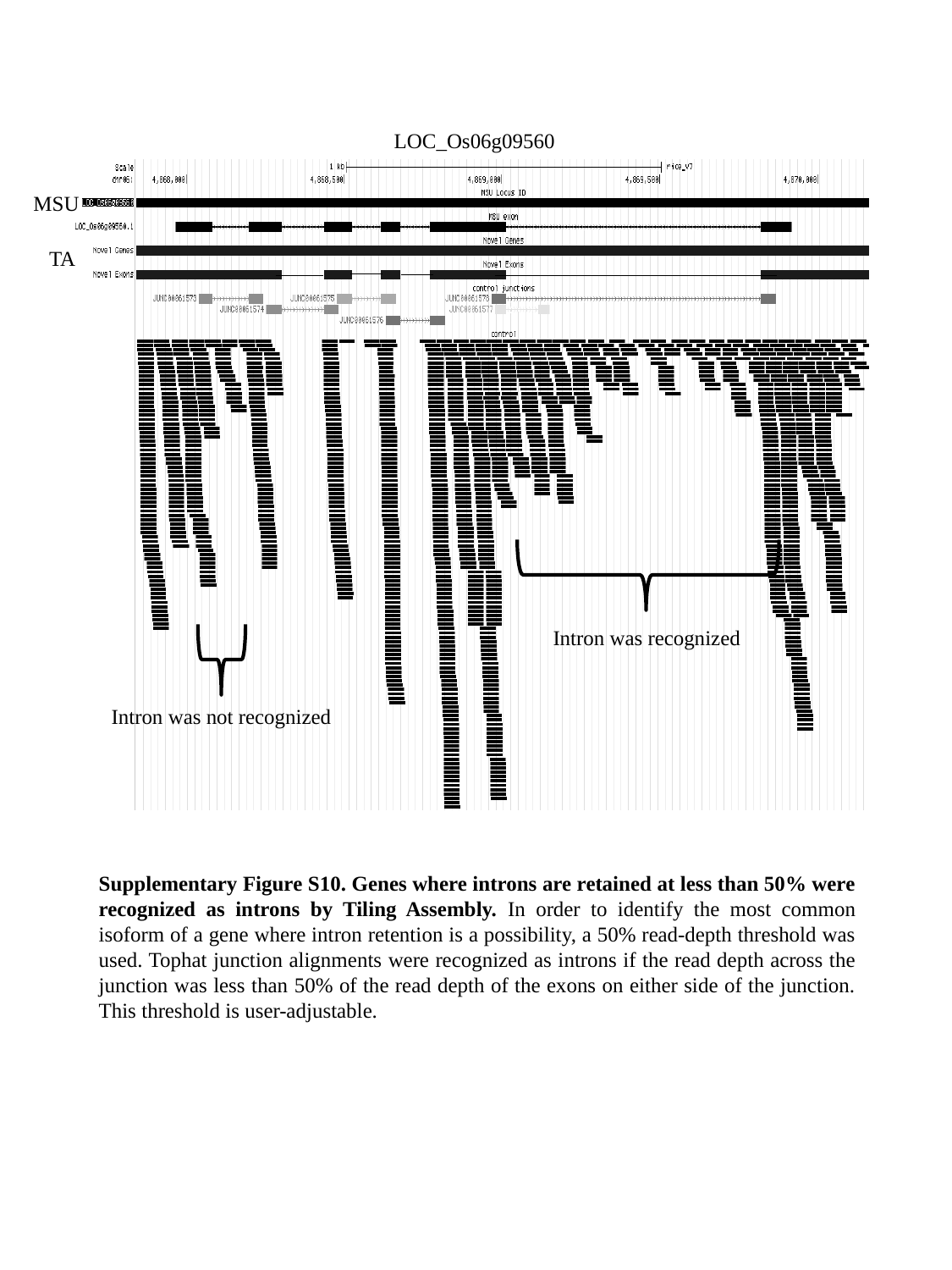

LOC_Os06g09560
MSU
TA
Intron was recognized
Intron was not recognized
Supplementary Figure S10. Genes where introns are retained at less than 50% were recognized as introns by Tiling Assembly. In order to identify the most common isoform of a gene where intron retention is a possibility, a 50% read-depth threshold was used. Tophat junction alignments were recognized as introns if the read depth across the junction was less than 50% of the read depth of the exons on either side of the junction. This threshold is user-adjustable.

## Slide 11
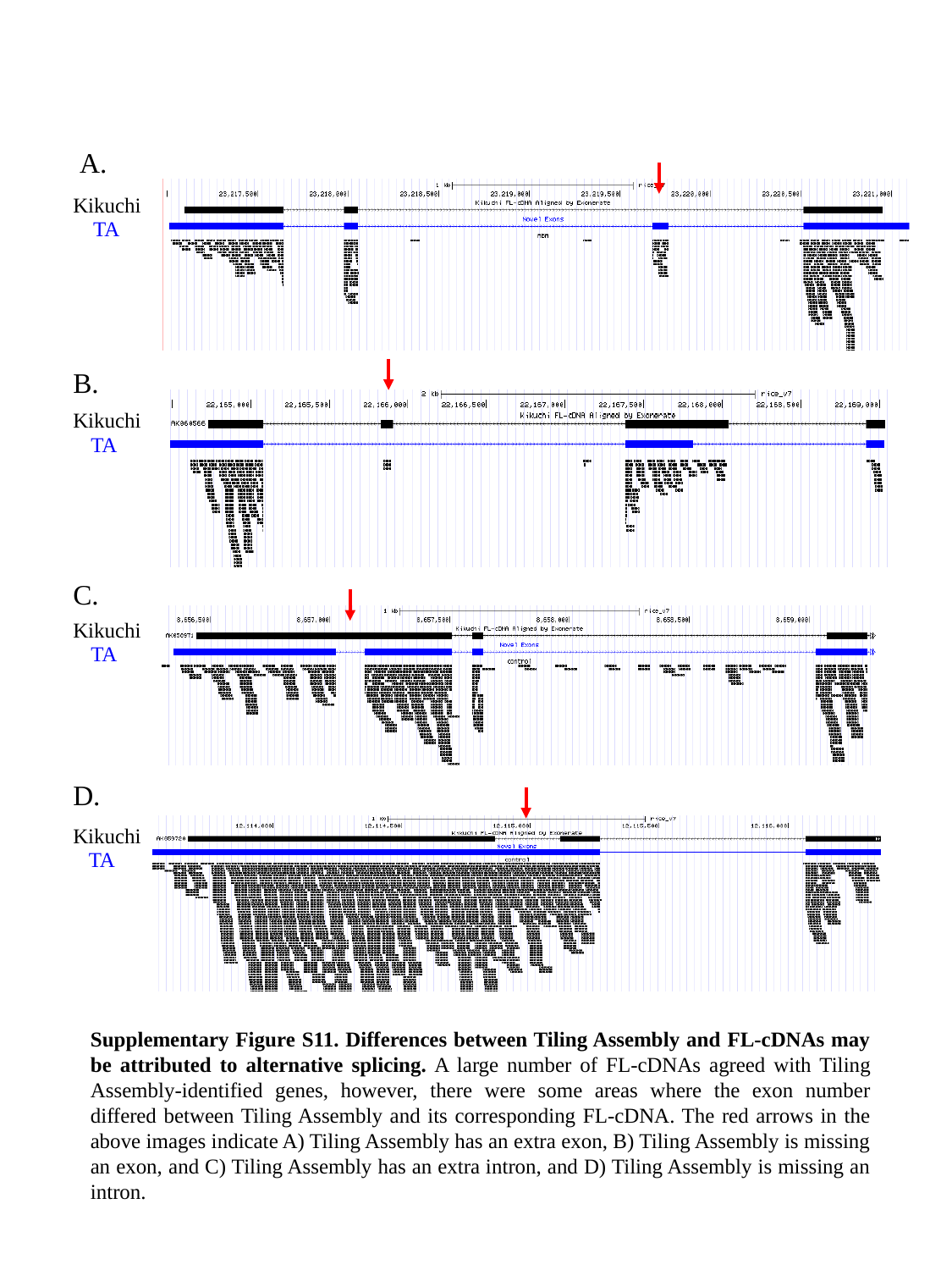

A.
Kikuchi
TA
B.
Kikuchi
TA
C.
Kikuchi
TA
D.
Kikuchi
TA
Supplementary Figure S11. Differences between Tiling Assembly and FL-cDNAs may be attributed to alternative splicing. A large number of FL-cDNAs agreed with Tiling Assembly-identified genes, however, there were some areas where the exon number differed between Tiling Assembly and its corresponding FL-cDNA. The red arrows in the above images indicate A) Tiling Assembly has an extra exon, B) Tiling Assembly is missing an exon, and C) Tiling Assembly has an extra intron, and D) Tiling Assembly is missing an intron.
